# Bowdoin Medical College

**Published:** 1871-12

**Authors:** 


### Bowdoin Medical College.

We take pleasure in announcing that our friend, Prof. E. W. Jenks, has accepted for one year the chair of Obstetrics and Diseases of Women and Children in Bowdoin Medical College. The performance of his new duties will in no way interfere with the Doctor's relations as President or Professor in the Detroit Medical College.

